# Draft Genome Assemblies of Two Campylobacter novaezeelandiae and Four Unclassified Thermophilic *Campylobacter* Isolates from Canadian Agricultural Surface Water

**DOI:** 10.1128/MRA.00249-21

**Published:** 2021-04-29

**Authors:** Mirena Ivanova, Bonnie Oh, Izhar U. H. Khan, Kendra Nightingale, Marie Bugarel, Amanda M. V. Brown, Guy H. Loneragan

**Affiliations:** a Animal and Food Sciences, Texas Tech University, Lubbock, Texas, USA; b Microbiological Sciences, Texas Department of State Health Services, Austin, Texas, USA; c Ottawa Research and Development Centre, Agriculture and Agri-Food Canada, Ottawa, Ontario, Canada; d Department of Biological Sciences, Texas Tech University, Lubbock, Texas, USA; e School of Veterinary Medicine, Texas Tech University, Amarillo, Texas, USA; University of Maryland School of Medicine

## Abstract

This report presents the draft genome sequences of two *Campylobacter novaezeelandiae* and four unclassified *Campylobacter* isolates from Canadian agricultural surface water. Phylogenomic analysis revealed that the six isolates formed unique clades, closely related to the disease-causing species C. jejuni, C. coli, and *C. hepaticus*.

## ANNOUNCEMENT

Contaminated surface water can spread pathogens and pose a threat to human health through a variety of exposure routes. Unclassified thermophilic *Campylobacter* spp. have been frequently recovered from various surface water types and sources in Canada (1–4). Here, six *Campylobacter* sp. isolates recovered between 2014 and 2015 from the surface water of two agricultural watersheds located at the South Nation River Basin east of Ottawa, Ontario (5), were subjected to whole-genome sequencing (WGS). Water samples were filtered through 0.47-μm membranes and the filters incubated in modified Preston broth (Oxoid, Lenexa, KS, USA) containing polymyxin B, rifampin, trimethoprim, and amphotericin B at 42°C under microaerophilic conditions (5% O_2_, 85% N_2_, and 10% CO_2_) for 48 h. The enriched samples were streaked onto modified Karmali agar (Oxoid) containing the same antibiotic supplement and incubated under the above-mentioned conditions (6). For WGS, the isolates were grown on modified *Campylobacter* blood-free selective agar (Thermo Fisher Scientific) or BD BBL Trypticase soy agar with 5% sheep blood (Thermo Fisher Scientific) under the same conditions described above. Biomass from the isolated colonies was subjected to genomic DNA extraction using the GenElute bacterial genomic DNA kit (Sigma-Aldrich) or the MagNA Pure LC total nucleic acid isolation kit (Roche). Nextera XT v2 libraries were prepared and 2 × 250-bp MiSeq Illumina reads generated. The paired-end reads were trimmed using Trimmomatic v0.38 (7) (-phred33). The quality of the sequence data before and after trimming was assessed using FastQC v0.11.8 (8). The trimmed reads were assembled using metaSPAdes v3.13.0 (k-mers 65, 77, 99, and 115) (9) and annotated using the Prokaryotic Genome Annotation Pipeline v4.2 (10). The quality of the assemblies was evaluated by Quast v5.0.2 (11) (Table 1). The average nucleotide identity (ANIb) was calculated using pyani v0.2.9 (12), and *in silico* DNA-DNA hybridization (isDDH) estimates were determined using the Genome-to-Genome Distance Calculator v2.1, formula 2 (http://ggdc.dsmz.de/ggdc.php). GFF files produced by Prokka v1.13.3 (13) were used as input for Roary v3.13.0 (14) to generate a core gene alignment at 55% protein identity cutoff (parameter -i 55). Gblocks v0.91b (15) was used to remove ambiguous alignments and phylogenetically uninformative positions (parameters: minimum length of a block was set to minimum, and positions where at least 50% of the sequences had a gap were treated as gap positions). The maximum-likelihood method in RAxML-NG v0.9.0 (16) was used to infer a phylogenetic tree from the alignment under the GTR+G substitution model with 100 bootstrap replicates. Default parameters were used for all software except where otherwise stated.

**TABLE 1:** Characteristics and accession numbers of the six thermophilic *Campylobacter* genome sequences

Parameter	Data for strain:					
	*Campylobacter* sp. CW4087 (2018MI34)	*Campylobacter* sp. CW4409 (TTU-622)	*Campylobacter* sp. CW4516 (TTU_617)	*C. novaezeelandiae* CW4519 (TTU_619)	*C. novaezeelandiae* CW4600 (TTU_621)	*Campylobacter* sp. CW4073 (2018MI35)
No. of reads	1,476,472	205,168	916,870	1,195,800	164,400	1,003,376
No. of bases (Mbp)	333.4	47.5	220.8	266.6	34.4	233
No. of contigs (≥500 bp)	80	215	49	123	369	51
k-mer coverage (×)	108	14	69	84	11	77
Nucleotide coverage (×)^*a*^	199	26	127	154	20	142
Genome assembly size (bp) (contigs ≥ 500 bp)	1,487,666	1,540,934	1,483,241	1,494,070	1,419,279	1,504,203
*N*_50_ (bp)	52,357	15,455	115,198	44,342	6,180	53,282
No. of CDSs (total)^*b*^	1,711	1,700	1,495	1,559	1,701	1,589
GC content (%)	26.81	26.75	26.73	27.44	27.65	28.32
No. of 16S rRNA genes	1	1	1	1	1	2
No. of 23S rRNA genes	3	1	1	1	1	1
No. of tRNA genes	40	39	39	39	39	40
No. of ncRNA genes^*c*^	3	3	3	3	3	3
No. of CRISPR/Cas genes	1					
SRA accession no.	SRR13349625	SRR13349624	SRR10695724	SRR10084915	SRR9894734	SRR13349623
GenBank accession no.	JAENKS000000000.1	JAENKR000000000.1	JAENKV000000000.1	JAENKU000000000.1	JAENKT000000000.1	JAENKQ000000000.1
BioProject accession no.	PRJNA686218	PRJNA686218	PRJNA296704	PRJNA296704	PRJNA296704	PRJNA686218
BioSample accession no.	SAMN17119060	SAMN17119061	SAMN13569984	SAMN12715343	SAMN12476126	SAMN17119062

a Nucleotide coverage = (*K*_mer_ coverage × read length)/(read length – *K*_mer_ length + 1), where *K*_mer_ length is the length of the longest k-mer used for the assembly (115 bp).

b CDSs, coding DNA sequences.

c ncRNA, noncoding RNA.

**FIG 1:**
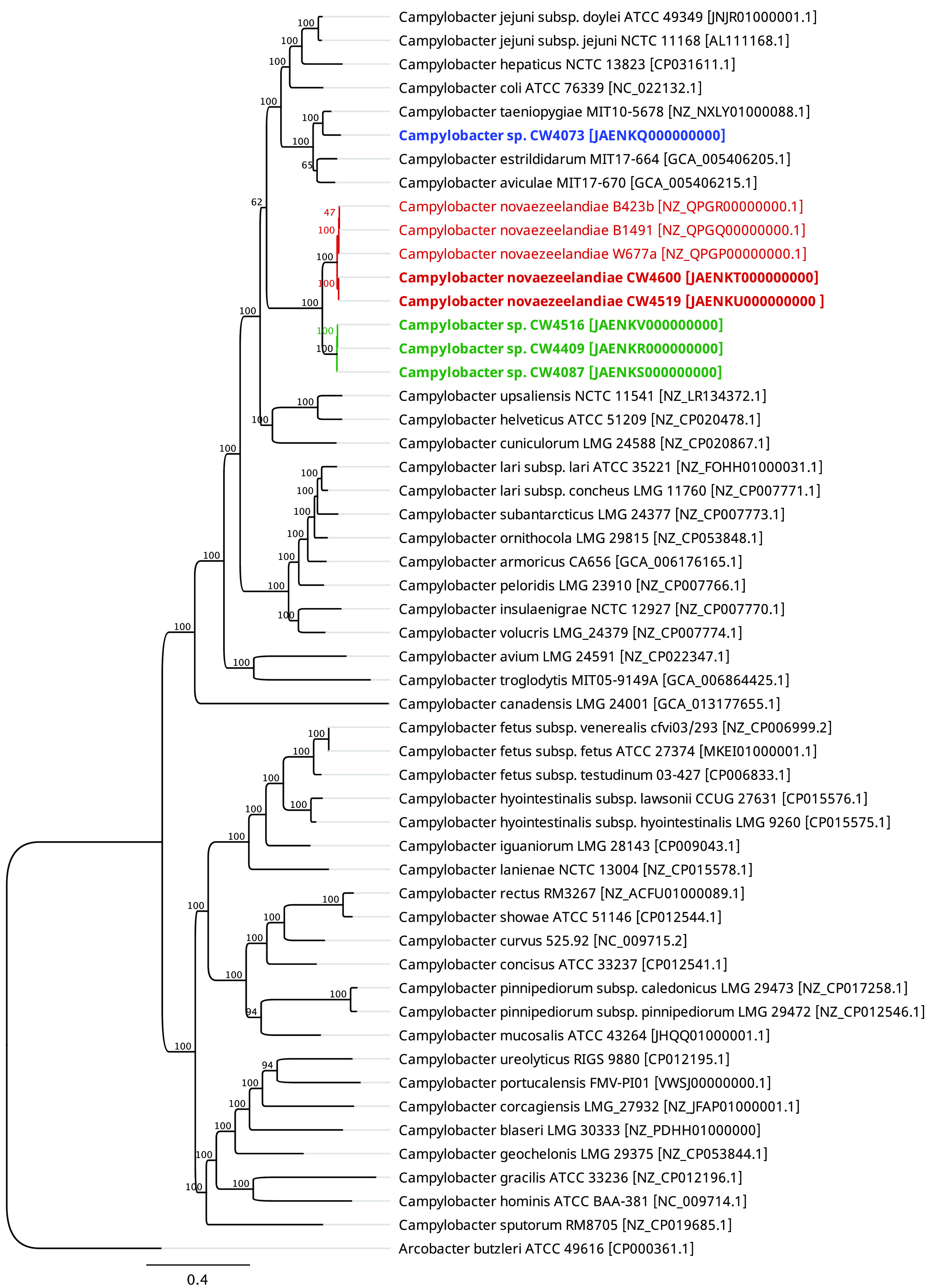
Maximum-likelihood phylogenetic tree showing the placement of the two Canadian *C. novaezeelandiae* (in red bold font) and the four unclassified *Campylobacter* (in blue and green bold font) isolates described in this study within the 38 currently known *Campylobacter* species. The tree was based on the alignment of 135 core genes (110 kb) and generated using the RAxML GTR+G substitution model with 100 bootstrap replicates. Geneious Prime v2020.0.4 (Biomatters, Ltd.) was used to visualize the tree. Arcobacter butzleri ATCC 49616 was used as an outgroup and to root the tree.

The *Campylobacter* sp. isolates clustered into three phylogenomic groups (Fig. 1). Strains CW4519 and CW4600 were identified as *C. novaezeelandiae*, having 98% ANIb and 84.1% isDDH to *C. novaezeelandiae* isolated in New Zealand (17). *Campylobacter* sp. strain CW4073 had *C. taeniopygiae* MIT10-5678 as its closest relative (91% ANIb, 42.3% isDDH), while *Campylobacter* sp. strains CW4087, CW4409, and CW4516 formed a unique cluster closest to *C. novaezeelandiae* (91% ANIb, 40.8% isDDH). The latter strains had the lowest GC content (26.8% ± 0.04% standard deviation [SD]) and genome size (1.50 Mbp ± 0.03 Mbp SD) among the *Campylobacter* spp. In all genomes, *bla*_OXA_ gene variants commonly found in *Campylobacter* were identified. The lack of other antimicrobial resistance genes would suggest that these isolates are commensally prevalent in the environment, with a low health risk to humans and animals.

### Data availability.

Isolates CW4409, CW4516, CW4519, and CW4600 were sequenced as part of the GenomeTrakr Project at the Texas Department of State Health Services (Austin, TX), and isolates CW4087 and CW4073 were sequenced at the Center for Biotechnology & Genomics, Texas Tech University (Lubbock, TX). Alignment and newick files were deposited into the Dryad Digital Repository (https://doi.org/10.5061/dryad.qrfj6q5f2).
